# Decadal Trends and Associated Factors of Colorectal Cancer in Kerala Between 2010 and 2021 Among Patients in a Tertiary Hospital-Based Cancer Registry

**DOI:** 10.7759/cureus.75030

**Published:** 2024-12-03

**Authors:** Ajayakumar Sunanda Vishakan, Rajan Akshaya, Priya Nair, Rohith Suresh, Roopa Paulose, Ajil Shaji, Brilly Michael Rose, Chippy Elizabeth Jose, Aswathy Sreedevi

**Affiliations:** 1 Community Medicine, Amrita Vishwa Vidyapeetham, Amrita Institute of Medical Sciences, Kochi, IND; 2 Gastroenterology and Hepatology, Amrita Vishwa Vidyapeetham, Amrita Institute of Medical Sciences, Kochi, IND; 3 Pathology, Amrita Vishwa Vidyapeetham, Amrita Institute of Medical Sciences, Kochi, IND; 4 Cancer Registry, Amrita Vishwa Vidyapeetham, Amrita Institute of Medical Sciences, Kochi, IND

**Keywords:** adenocarcinoma, colorectal cancer, histology, hospital-based cancer registry, kerala, secondary data analysis

## Abstract

Introduction

The incidence of colorectal cancer (CRC) has notably risen on a global scale, owing to modifications in lifestyle patterns and the overconsumption of processed food to meet nutritional requirements. Colorectal cancer is among the most common cancers in India, with a significant number of new cases reported annually. The aim of our study is to examine the trends and association of CRC in Kerala between the years 2010-2021 among patients reporting at a tertiary Hospital-Based Cancer Registry (HBCR) in Ernakulam.

Methods

A secondary data analysis was done using the data of CRC obtained from HBCR at Amrita Institute of Medical Sciences (AIMS), Kochi. All the reported patients diagnosed with CRC between the years 2010 and 2021 and residing in Kerala were included in the study. A total of 2,995 patients were included in the analysis.

Results

CRC cases rose until 2016 and then slightly decreased. However, joinpoint regression analysis showed a consistent rate of 0.1 cases per year, with no significant increase. Adenocarcinoma emerged as a histological subtype, and the highest number of CRC cases was reported in the year 2018; the prevailing and lowest was reported in the year 2020. Age and sex showed statistically significant associations with the histology of CRC. Furthermore, associations were also seen between the site of the lesion and the age of the patient.

Conclusion

The trends of CRCs showed that the cases were stable at a rate of 0.1 per year, and there was no significant increase. Robust cancer registries are required to continuously monitor CRC trends and outcomes, facilitating evidence-based decision-making.

## Introduction

Colorectal cancer (CRC) is the third most commonly diagnosed cancer in the world after breast and lung cancer. Many factors, such as genetic and environmental factors, contribute to the development of the disease [1]. CRC presents a distinct concern due to its status as the second leading cause of cancer-related mortality after lung cancer [2]. Globally, in the year 2022, approximately 1.93 million individuals were diagnosed with CRC, with over 900,000 deaths, highlighting the immense burden on the healthcare system and communities worldwide [2].

As per the study conducted by Mathew et al., trends of CRC incidence rates in India from 2004 to 2014 have increased by 20.6%, affecting younger individuals under 50 years of age [3-5]. Despite its impact on disability-adjusted life years, as the sixth leading cause of years lost to disability in India, CRC has not been included in government-led screening programs [6]. In Kerala, government-run hospitals, such as the Regional Cancer Centre Thiruvananthapuram (RCC TVM), have reported increasing CRC cases over the years [7]. The awareness of CRC, its risk factors, and the willingness to undergo screening are comparatively low in the Indian population. The reasons for delay in seeking medical care may be due to the asymptomatic initial phase of CRC, fear of screening colonoscopy, and stigma faced by cancer patients. Additionally, there is a significant gap in the availability of safe and high-quality endoscopy services, which poses a major challenge in the early detection and diagnosis of CRC, particularly in resource-limited settings [8,9]. Around a quarter of patients are diagnosed with metastasis at presentation, and nearly half of those with CRC will eventually develop metastasis [10,11].

Risk factors for CRC can be modifiable or non-modifiable; modifiable risk factors include obesity, type 2 diabetes mellitus, specific dietary choices, smoking, and alcohol consumption [12,13]. Non-modifiable risk factors include age, racial and ethnic background, sex assigned at birth, family history, inherited syndromes (Lynch syndrome, hereditary non-polyposis CRC, and familial adenomatous polyposis), history of certain medical conditions like colorectal polyps, and inflammatory bowel disease, the latter comprising ulcerative colitis and Crohn’s disease [14]. The increasing incidence seen recently may be due to the adoption of Western lifestyles among the Asian population [14]. Kerala is a state where meat consumption is the highest, which is a risk factor for developing CRC [15,16]. Non-modifiable risk factors highlight the need for early screening in high-risk individuals. Regular colonoscopies are critical, especially for those over 50 years of age [17]. Screening is also advised for people under 50 years of age if they have any specific risk factors or a family history of cancer.

There is a dearth of studies examining the trends and associations of CRC, including the relationship between the primary site of the lesion and sociodemographic characteristics in Kerala. This study aims to find the trends and associations of CRC in Kerala over a decade (between the years 2010 and 2021) among patients included in a hospital-based cancer registry (HBCR).

## Materials and methods

A secondary data analysis was done using the HBCR data of Amrita Institute of Medical Sciences, Kochi, Kerala. Necessary permissions were obtained from the institution for data retrieval. A total data of 3,281 patients were obtained, of which 286 patients were excluded as histology was not clearly mentioned.

Inclusion criteria

All patients residing in Kerala diagnosed with CRC from the year 2010 to 2021 registered in the HBCR of Amrita Institute of Medical Sciences were included in the study.

Exclusion criteria

Patients with CRC histology that was not specified, such as “unknown,” “malignancy,” or “neoplasm,” were excluded from the study.

The data were obtained in MS Excel (Microsoft Corp., Redmond, WA, US) and analyzed using Jamovi 2.4 software (https://www.jamovi.org). Continuous variables were expressed as the mean and standard deviation, and categorical variables were expressed as frequency and percentage. The specific histology of the malignancy and the topography of the lesions were classified according to standard guidelines. Topography was classified as “ascending colon,” “descending colon,” “rectum,” and “others.” “Others” include carcinoma in the appendix, cecum, hepatic flexure, transverse colon, splenic flexure, recto-sigmoidal, sigmoidal, and colon not otherwise specified.

The trend of CRC cases over the last 12 years has been assessed using Joinpoint regression software version 5.1.0 (National Cancer Institute, Bethesda, MD, US), and the number of CRC cases was converted to log values for doing the regression model. The analysis employed the best-fit piecewise continuous log-linear model to identify significant changes in trends over time. A maximum of three joinpoints was allowed, ensuring a sufficient number of data points between joinpoints for model stability. Statistical significance was assessed using the Monte Carlo permutation method with 4,499 permutations, a significance level of 0.05, and 95% confidence intervals (CIs). The annual percentage change (APC) was calculated for each segment to determine the magnitude and direction of trends. Graphical outputs and tabular data displaying APCs, p-values, and CIs were generated for comprehensive interpretation.

The association between the histology of CRC and the site of the lesion and sociodemographic characteristics was assessed using the chi-squared test. A p-value of less than 0.05 was considered statistically significant. Multivariable logistic regression modeling was done using independent variables for p-value <0.2 in univariate analysis. Binomial logistic regression was done between the histology of CRC with age and sex. Multinomial logistic regression was done between the topography of the lesion with age and sex.

## Results

A total of 2,995 patient data were obtained through the cancer registry between the years 2010 and 2021. The mean age of the persons with CRC was 61.5 ± 12.4 years. About 60.2% of CRC patients were aged 60 years and above, and more than half of the patients (59%) were men. Geographically, over half of the CRC cases reporting to the HBCR were from Central Kerala (51.3%), followed by South Kerala (42%). The most common histological presentation was adenocarcinoma, accounting for 96.7% of cases. More than half (54.3%) of the CRC was situated in the rectum, followed by descending colon (30.5%) and ascending colon (12.8%) (Table 1).

**Table 1:** Distribution of study participants based on sociodemographic features, histology, and site of lesion (n = 2,995).

	Variables	Frequency (n)	Percentage (%)
Age (in years)	<60	1,191	39.8
	≥60	1,804	60.2
Sex	Female	1,227	41.0
	Male	1,768	59.0
District	Central Kerala	1,536	51.3
	North Kerala	200	6.7
	South Kerala	1,259	42.0
Histology	Adenocarcinoma	2,896	96.7
	Lymphomas	32	1.1
	Neuroendocrine tumors	25	0.8
	Others	42	1.4
Topography	Ascending colon	384	12.8
	Descending colon	916	30.6
	Other	69	2.3
	Rectum	1,626	54.3

**Figure 1:**
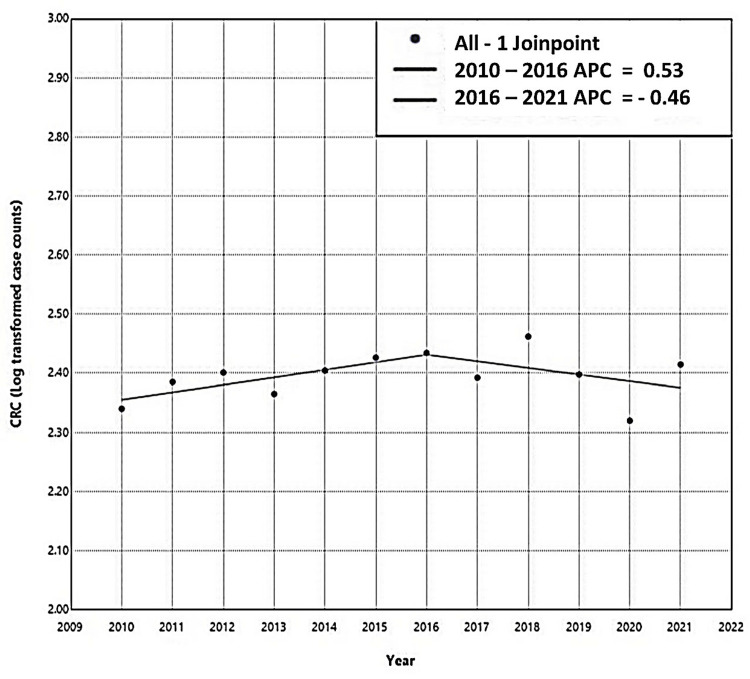
Joinpoint regression analysis showing decadal trends of colorectal cancer reported at the tertiary care center. CRC: colorectal cancer; APC: annual percentage change

As per the HBCR data, the highest number of 290 cases was in the year 2018, and the lowest in 2020, 209 patients. The absolute number of CRCs seems to have increased up till the year 2016 with a slight decline thereafter. The joinpoint regression analysis of CRC case counts (log-transformed) from 2010 to 2021 shows an increasing trend from 2010 to 2016, with an APC of +0.53%, followed by a decreasing trend from 2016 to 2021, with an APC of -0.46%. However, neither of these trends was statistically significant. Overall, from 2010 to 2021, the cases stabilized at an approximate rate of 0.1% per year, though this stabilization also lacked statistical significance (p-value, 0.55) (Figure 1).

Adenocarcinoma was the predominant histological subtype of CRC in both sexes. Among people aged 60 years and above, 97.8% had adenocarcinoma; the proportion was significantly higher as compared to 95% under 60 years of age (p-value <0.001). This histological subtype was common in all three regions of Kerala (Table 2). A statistically significant association was observed between age and topography of CRC. The rectum seemed to be more commonly affected among all the age groups, as compared to other sites of the lesion (p-value, 0.011). Therefore, irrespective of the location of the tumor in the colon, it was more common in people over age 60 (Table 2).

**Table 2:** Univariate analysis showing the association of age, sex, and place of residence with histology and topography of colorectal cancer (n = 2,995). The chi-squared test was used for analysis, *statistically significant, p < 0.05 CRC: colorectal cancer

	Histology of CRC			Topography of lesion				
	Adenocarcinoma	Others	p-value	Rectum	Descending colon	Ascending colon	Others	p-value
Age			<0.001*					0.011*
<60	1,132 (95%)	59 (5%)		660 (55.4%)	340 (28.5%)	152 (12.8%)	39 (3.3%)	
≥60	1,764 (97.8%)	40 (2.2%)		966 (53.5%)	576 (31.9%)	232 (12.9%)	30 (1.7%)	
Sex			0.075					0.21
Female	1,195 (97.4%)	32 (2.6%)		658 (53.6%)	391 (31.9%)	157 (12.8%)	21 (1.7%)	
Male	1,701 (96.2%)	67 (3.8%)		968 (54.8%)	525 (29.7%)	227 (12.8%)	48 (2.7%)	
Region			0.473					0.606
Central	1,490 (97%)	46 (3%)		835 (54.4%)	474 (30.9%)	191 (12.4%)	36 (2.3%)	
North	191 (95.5%)	9 (4.5%)		111 (55.5%)	59 (29.5%)	22 (11.0%)	8 (4%)	
South	1,215 (96.5%)	44 (3.5%)		680 (54.0%)	383 (30.4%)	171 (13.6%)	25 (2.0%)	

Multivariable logistic regression was done to find the independent predictors associated with the histology of CRC. These models showed that the elderly age group, more than or equal to 60 years of age, had more than 2.38 times the odds of developing adenocarcinoma as compared to other histological subtypes of CRC. It was also seen that female patients had 1.58 times the risk of developing adenocarcinoma as compared to male patients (Table 3).

**Table 3:** Multivariable logistic regression. Independent predictors of colorectal adenocarcinoma. AOR: adjusted odds ratio; CI: confidence interval

Independent variables		AOR (95% CI)	p-value
Age	<60	1	<0.001
	≥60	2.38 (1.58-3.59)	
Sex	Male	1	0.036
	Female	1.58 (1.03-2.43)	

The multivariable multinomial logistic regression analysis shows that individuals aged 60 years or older have significantly higher odds of developing CRC in the descending colon followed by ascending colon and rectum at 2.31 (95% CI 1.41-3.8), 2.07 (95% CI 1.23-3.84), and 1.98 (95% CI 1.21-3.22) times, respectively, compared to “other sites” with p-values all below 0.01. Regarding sex, female patients have higher odds of cancer in the descending colon (adjusted odds ratio (AOR): 1.83, p = 0.026) compared to “other sites” than male patients. However, while female patients also have higher odds of rectal (AOR: 1.65, p = 0.062) and ascending colon (AOR: 1.68, p = 0.066) cancer, these associations are not statistically significant (Table 4).

**Table 4:** Multivariable multinomial logistic regression of predictors of topography of colorectal cancer. AOR: adjusted odds ratio; CI: confidence interval

Independent variables		Descending colon		Rectum		Ascending colon	
		AOR (95% CI)	p-value	AOR (95% CI)	p-value	AOR (95% CI)	p-value
Age	<60	1	<0.001	1	0.006	1	0.006
	≥60	2.31 (1.406-3.80)		1.98 (1.215-3.22)		2.07 (1.229-3.84)	
Sex	Male	1	0.026	1	0.062	1	0.066
	Female	1.83 (1.074-3.11)		1.65 (0.975-2.78)		1.68 (0.966-2.93)	

## Discussion

The analysis of 2,995 CRC cases from the HBCR spanning from 2010 to 2021 reveals that the cases were stable at a rate of 0.1 per year. Adenocarcinoma was the most prevalent histological subtype, comprising 96.7% of cases, and was more common among patients aged 60 and above (97.8%). The rectum was the most frequently affected site (54.3%), followed by the descending colon (30.5%) and the ascending colon (12.8%). The odds of developing malignancies at all sites were higher among those aged 60 years and above. Female patients are more likely than male patients to develop cancer in the descending colon compared to “other sites.”

The annual reports of RCC TVM indicated a steady increase in CRC cases from 625 in 2011 to 826 in 2021, marking a rise of 201 cases over the decade [7]. A substantial drop was observed in 2020 in both our center and RCC TVM due to the COVID-19 pandemic, with a subsequent rebound in reported cases. The higher numbers in RCC TVM may be due to higher patient volume in the public sector compared to our private institution.

Our analysis revealed that 60.2 percent of patients were aged more than 60 years, with a mean age of 61.5 years, with ages ranging from 13 to 95 years. In contrast, a study done by Patil et al. at Tata Memorial Hospital, Mumbai, reported the mean age of patients to be 47.2 years with a range of 11-85 years. The study by Patil et al. was conducted only for a period of two years, which could have accounted for the lower mean age [18]. In a study in the United States [19], 89% of colorectal carcinomas were above 50 years of age.

The HBCR analysis of our institute showed that CRCs were seen more among male patients; similar results were also seen in studies done in other parts of the country [7,18,20-22]. The majority of CRCs reported in the cancer registry are concentrated in Central Kerala (51.3%), followed by South Kerala (42%). This geographical distribution may be attributed to the proximity of our institution to this region.

In this study, it was found that adenocarcinoma was the most common histological type of CRC, accounting for 96.7%; this aligns with global trends where adenocarcinoma is the dominant form of CRC [23,24]. The rectum was the most common site of lesion, affecting 54.3% of patients, respectively. Several other studies also reported similar results [12,18,20-22,25] without any geographical differences.

Individuals aged 60 and older were more frequently diagnosed with adenocarcinoma compared to other histological types. Moreover, the risk of developing adenocarcinoma was higher in female patients than in male patients. Further studies are needed to confirm this finding. However, it is worth noting that the CI for the AOR is nearing 1. In contrast, other research has typically shown that male patients are more likely than female patients to develop adenocarcinoma [26,27]. The number of CRC cases mildly fluctuated over the study period, with no statistically significant increase. However, longer-term studies might be necessary to detect temporal trends.

As far as the investigators are aware, there are limited studies in India that examine the trends of CRC and its correlation with demographic factors. Our study fills this gap by encompassing a significant time frame from 2010 to 2021. Trend analysis significance was done using Joinpoint software, which was developed by the National Cancer Institute.

The limitation of this is that, as it relied on secondary data, there were only a limited number of variables available for analysis. Consequently, we could not investigate other factors such as diet or personal habits. Additionally, since this HBCR is based in a private institution, the findings may not be generalizable to the broader population.

## Conclusions

This study provides valuable insights into the characteristics of CRC patients in Kerala. Trend analysis showed that the cases were stable at an APC of 0.1 cases per year, and there was no significant increase. Adenocarcinoma is the dominant histological type, with the rectum as the most common primary site. Age is a significant risk factor, especially after 60 years of age, highlighting the need to implement CRC screening through the National Program for Health Care of the Elderly. Further research is required to explore the underlying causes of CRC in Kerala, particularly with respect to behavioral and lifestyle risk factors. Additionally, capturing comprehensive data on risk factors, the stage of diagnosis, and patient outcomes, including survival in the cancer registry, can aid in shaping policies and strengthen public health efforts in managing CRC effectively.
